# Hydrogen Sulfide Improves Postharvest Quality of Okra (*Abelmoschus esculentus* (L.) Moench) Pods by Enhancing Antioxidant Capacity and Delaying Lignification

**DOI:** 10.3390/foods13162617

**Published:** 2024-08-21

**Authors:** Weihua Luo, Tinghui Chen, Xiao Gong, Jingjing Chen, Wei Zhou, Jihua Li

**Affiliations:** 1 Key Laboratory of Tropical Crop Products Processing Ministry of Agriculture and Rural Affairs, Agricultural Products Processing Research Institute, Chinese Academy of Tropical Agricultural Sciences, Zhanjiang 524001, China; b2000lwh@163.com (W.L.); jgscth@163.com (T.C.); weizhou111@foxmail.com (W.Z.); foodpaper@126.com (J.L.); 2 College of Food Science & Technology, Huazhong Agricultural University, Wuhan 430070, China; 3 Key Laboratory of Tropical Fruit Biology, Ministry of Agriculture and Rural Affairs, South Subtropical Crops Research Institute, Chinese Academy of Tropical Agricultural Sciences, Zhanjiang 524091, China; chenjingjing0704@163.com

**Keywords:** okra pods, hydrogen sulfide (H_2_S), antioxidant enzymes, lignification, storage characters

## Abstract

Okra (*Abelmoschus esculentus* (L.) Moench) pod storage is challenging due to its high water content and tendency to lignify. Sodium hydrosulfide (NaHS) served as an H_2_S donor in this investigation. Compared with the control group, the group treated with 0.5 mmol/L NaHS solution effectively maintained the appearance quality, and its weight loss was only 6.21% at 20 days. The H_2_S treatment not only preserved tissue nutrients but also significantly enhanced catalase (CAT), ascorbic acid peroxidase (APX), and superoxide dismutase (SOD) activities while decreasing oxidant damage. In addition, H_2_S slowed down lignin synthesis by inhibiting the activities of key enzymes such as phenylalanine ammonialyase (PAL), cinnamate 4-hydroxylase (C4H), and cinnamyl alcohol dehydrogenase (CAD) in the lignin biosynthesis pathway. Transcriptome analysis revealed that H_2_S affects 34 genes in the phenylpropanoid biosynthesis pathway, such as *AePAL*, *Ae4CL1*, *AeCCOAOMT1*, *AePOD*, etc., which inhibit lignin synthesis of okra pods. All in all, moderate H_2_S can improve postharvest quality and extend the shelf-life of okra pods by enhancing antioxidant capacity and delaying lignification; the results will provide an overview of its application in the preservation of okra pods.

## 1. Introduction

Okra (*Abelmoschus esculentus* (L.) Moench), an annual plant belonging to the Mallow family, features pod-shaped fruits and is also known as lady’s finger. Okra was discovered in India and then introduced to tropical and temperate countries in Asia, Africa, and North America. The cultivation of okra is very important to the local economy [1]. As a tropical vegetable, okra pods are typically harvested in the summer. It is common to water loss and shrinkage due to its large surface area and vigorous respiration, leading to a short shelf life [2]. Also, okra is prone to issues such as lignification, browning, softness, and nutrient loss during storage, all of which negatively impact its flavor. These challenges have diminished the nutritional value of okra and limited the development of the industry.

The imbalance of reactive oxygen species (ROS) and antioxidant scavenging system was closely related to the postharvest aging of fruits and vegetables. Excessive reactive oxygen species would accelerate membrane lipid peroxidation and then destroy cell structure. The antioxidant defense system is made up of antioxidants and antioxidant enzymes in which enzymes such as superoxide dismutase (SOD), catalase (CAT), and ascorbate peroxidase (APX) can be activated for the scavenging of reactive oxygen species, thereby reducing cell membrane damage [3]. Lignification is an important index affecting the quality of okra, mainly due to the accumulation of lignin content. The major enzymes in the lignin biosynthesis pathway include phenylalanine ammonialyase (PAL), cinnamate 4-hydroxylase (C4H), 4-coumarate:CoA ligase (4CL), and cinnamyl alcohol dehydrogenase (CAD) [4]. Therefore, to preserve the quality of postharvest okra, it is critical to strengthen the antioxidant defense system and reduce the activity of lignin biosynthesisase. 

Hydrogen sulfide (H_2_S), considered the third endogenous gas signaling molecule, alongside carbon monoxide and nitric oxide [5], has been extensively studied in the field of biomedicine for its anti-inflammation, anti-aging, and anti-oxidation properties [6,7]. Currently, sodium hydrosulfide (NaHS) is used as an H_2_S donor to preserve agricultural products through solution impregnation or gas fumigation [8,9]. Gas fumigation technology offers advantages over solution impregnation in terms of convenience and high permeability [10]. In recent years, H_2_S has been used for delaying the ripening and senescence and extending the shelf life of fresh fruits and vegetables, such as button mushrooms [8], goji berry [9], pointed gourd [11], peaches [12], zucchini [13], etc. The primary mechanism aims to maintain the quality of products by regulating respiration and ethylene synthesis [8], increasing the activity of the reactive oxygen scavenging system enzyme [14], and combating microbial infection [15]. The impact of H_2_S on lignification metabolism has been reported in pointed gourd [11]. Hypothesize that the lignification process is mostly responsible for the decline in okra quality. However, the role of H_2_S in postharvest storage of okra has not been reported, and the mechanism of H_2_S is still unclear.

Transcriptome sequencing (RNA-Seq) technology is defined as a sequencing technology that acquires the genome of a target sample. The transcription database of the various okra organs was produced by Zhang et al. [16], and it contains valuable information about the biosynthesis pathway of flavonoids and polysaccharides. Zhan et al. [17] found that melatonin can enhance the tolerance of okra to salt stress. RNA-Seq technology can also use transcription data to examine the structure and function of significant genes. Furthermore, the molecular mechanisms pertaining to the preservation of fruits and vegetables have been obtained through the analysis of metabolic pathways enriched by differentially expressed genes (DEGs) in recent years [18]. This is a crucial development for the molecular study of preservation experiments. However, transcriptome analysis of okra lignification has not been reported.

The purpose of this study is to investigate how H_2_S fumigation treatment affects okra pod storage quality, reactive oxygen metabolism, and lignification-related enzyme activities. Moreover, RNA-Seq technology was used to provide transcriptional information on okra after H_2_S treatment. The aim is to establish a scientific foundation for okra pod preservation by analyzing the effect of H_2_S on the postharvest storage quality of okra pods.

## 2. Materials and Methods

### 2.1. Experimental Design

Mature okra pods of cultivar ‘Yuehai’ (up to 9–12 cm in length, 1.5–2.0 cm in diameter) were gathered in August 2023 during the harvest season from Naozhou island (Zhanjiang city, Guangdong province, China) at 20°54′ N and 110°36′ E. The variety was certified by Sun Huaizhi of the Guangzhou Academy of Agricultural Sciences, and the approval number was Guangdong Audit Dish 2012008. The newly acquired materials were taken to the laboratory in the early morning and kept in a cool environment for 1 h to eliminate the field heat.

NaHS (NaHS·xH_2_O, 68–72%, purchased from Rhawn, Shanghai, China) was used as an H_2_S donor, which dissolves in water to rapidly release H_2_S gas. Distilled water was used as the control group, and NaHS solution was used as the treatment group. Various concentrations of NaHS solution (0, 0.25, 0.5, 0.75, 1.0, 1.5, and 2.0 mmol/L) were prepared in preservation boxes (volume 12 L). The optimum fumigation concentration for okra was 0.5 mmol/L. The NaHS solution was changed every 24 h. Okra pods were stored at 5 ± 1 °C and 65–75% RH for 20 d. To obtain a uniform sample, okra pods that were 4–6 cm in length from the head and seeds are not needed. Samples were ground into a powder under liquid nitrogen and kept in a refrigerator at a temperature of −80 °C until use.

### 2.2. Determination of Weight Loss Rate and Hardness

The weight loss rate was measured using the weighing method. The weights of samples were recorded at the same time every four days, and it was calculated on the basis of the following formula [15]:

Weight loss rate (%) = [(W_0_ − W_t_)/W_0_] × 100

where W_0_ is the weight at day 0, and W_t_ is the weight during storage.

The hardness of the okra pods was assessed utilizing a fruit firmness tester (GY-4, Zhiqu Precision Instruments Co., Ltd., Dongguan, China), which was equipped with a probe with a diameter of 3 mm. The probe was slowly pressed into the pod and stopped when the depth reached 5 mm. The three pods were measured, and the results were expressed as force measured in N.

### 2.3. Determination of Ascorbic Acid, Soluble Protein, Protopectin, and Soluble Pectin Contents

The 2,6-dichloroindophenol method was utilized to quantify the amount of ascorbic acid [14]. A 2.0 g sample of powder was thoroughly mixed with 30 mL of 20 g/L oxalic acid for 10 min. Following this, the mixture was centrifuged at a speed of 4000× *g* for 20 min to separate and collect the supernatant. An amount of 10 mL of supernatant was then titrated with a 2,6-dichloroindophenol solution. The result of the analysis was expressed in terms of mg/100 g.

The Bradford method was adopted to measure the soluble protein content, and modifications were made to the method [19]. Briefly, okra pod powder (0.2 g) was ground in 1 mL of distilled water, followed by centrifugation at 12,000× *g* and 4 °C for 20 min. An amount of 1 mL of 10-fold diluted supernatant was added to Coomassie Brilliant Blue G-250 5 mL; the mixed solution was thoroughly blended and left for 2 min. The content of soluble protein was read at 595 nm and calculated according to a standard curve made using bovine serum albumin. The final result is expressed as mg/g.

Protopectin and soluble pectin were determined using the modified method of Liu et al. [20]. Okra pod powder (1.0 g) was homogenized with 25 mL of 95% (*v*/*v*) ethanol after 30 min of boiling water bath, cool down, followed by centrifugation at 4000× *g* for 15 min. The steps were repeated three times after removing the supernatant. The precipitate was mixed with distilled water 20 mL and maintained for 30 min at 50 °C. The supernatant was collected as soluble pectin. The precipitate was dissolved in 12.5 mL of H_2_SO_4_ (0.5 mol/L) and reacted for 1 h in a boiling water bath. After cooling down, the supernatant was collected as protopectin. The absorption value at 530 nm was determined, and the result was expressed as a mass fraction of galacturonic acid (%).

### 2.4. Determination of Lignin and Cellulose Contents

The lignin content was measured using an improved method, as reported by Xie et al. [21]. Okra pod powder (1.0 g) was blended with 95% ethanol 10 mL and subsequently underwent centrifugation at 4000× *g* for 10 min. The mixed solution of 95% ethanol and ethanol/hexane = 1:2 (*v*/*v*) was used to rinse the precipitate, respectively. After being dried, the precipitate was dissolved in a water bath at 70 °C for 30 min using a 25% bromoacetyl-glacial acetic acid solution (2 mL). The solution was fixed to 10 mL with glacial acetic acid after sequentially adding 2 mol/L NaOH, glacial acetic acid, and 0.1 mol/L hydroxylamine hydrochloric acid. After centrifugation, the supernatant was diluted 30 times. The results of absorbance values at OD_280_ were described on a fresh weight basis (FW) as OD_280nm_/g.

The cellulose content was measured by modifying the Oomen et al. [22] method. After adding 2 mL of 50 mmol/L Tris/HCl (pH 7.2) solution to 0.2 g of powdered okra pods, the mixture was continuously shaken for 3 h at room temperature. Subsequently, the sample underwent centrifugation at 12,000× *g* for 10 min. The precipitate was rinsed with water, ethanol, and acetone, followed by drying. A 1 mL trifluoroacetic acid solution (2 mol/L) was combined with 10 mg of the sample, and it was incubated for 90 min at 120 °C. Following cooling, the sample underwent centrifugation for 10 min at 12,000× *g*, and the residual cellulose precipitate was cleaned with water and ethanol. The precipitate was treated with 67% (*v*/*v*) H_2_SO_4_ and placed in a water bath at 37 °C for 60 min. The content of cellulose was calculated from the glucose standard curve and expressed as mg/g.

### 2.5. Determination of Malondialdehyde (MDA), Superoxide Anion (O_2_^·−^), and Hydrogen Peroxide (H_2_O_2_) Contents

The determination of MDA was carried out strictly according to the method of the kit (Nanjing Jiancheng Bioengineering Institute Co., Ltd., Nanjing, China). The O_2_^·−^ content was measured using the kit (Beijing Solepol Science and Technology Co., Ltd., Beijing, China) in accordance with the manufacturer’s instructions.

With minor modifications, the H_2_O_2_ content was measured using Zhang et al. [23]. An amount of 1.5 mL of pre-cooled acetone was added to the okra pod powder (0.3 g) and ground to a homogeneous. Following this, the mixed solution underwent centrifugation for 20 min at 4 °C at 12,000× *g*. The supernatant (1 mL) was mixed with 0.1 mL of 10% titanium tetrachloride–hydrochloric acid solution and 0.2 mL of concentrated ammonia. After 5 min of reaction, the mixture underwent centrifugation for 15 min at 4 °C at 12,000× *g*. The precipitate was added to 3 mL of 2 mol/L sulphuric acid and shaken to dissolve the precipitate completely. The OD_412_ nm values of samples were recorded. The results were calculated from the standard curve and expressed as mmol/L.

### 2.6. Determination of Catalase (CAT), Ascorbic Acid Peroxidase (APX), and Superoxide Dismutase (SOD) Activities

The CAT activity of okra pods was measured using the method previously reported by Xu et al. [24] with minor adjustments. Okra pod powder (0.3 g) was mixed with 1 mL of buffer solution (containing 5% PVP, dissolved in 0.1 mol/L PBS with a pH of 7.5) and quickly ground to homogenate under ice bath conditions. Centrifugation at 12,000× *g* for 20 min at 4 °C produced the supernatant. The reaction mixture consisted of 2.9 mL H_2_O_2_ (20 mmol/L) and 0.1 mL the supernatant. After 15 s, the absorbance at 240 nm was measured to establish the reaction’s initial value. Subsequently, measurements were taken every 30 s for 3 min. The specific activity was expressed as U/(g·min) FW.

The APX activity was evaluated in accordance with Zhang et al. [23] method, with minor adaptations. An amount of 1.0 mL of pre-cooled 0.1 mol/L potassium phosphate buffer (pH 7.5, containing 0.1 mmol/L EDTA, 1 mmol/L ascorbic acid, and 2% PVPP) was mixed with 0.1 g of powdered okra pods under ice bath condition. The supernatant (0.1 mL) and pH 7.5 potassium phosphate buffer (2.6 mL, containing 0.1 mmol/L EDTA and 0.5 mmol/L ascorbic acid) were mixed, and 0.3 mL of 2 mmol/L H_2_O_2_ was added and mixed thoroughly. The initial absorption value at 290 nm was recorded for 15 s, followed by recordings every 30 s for 3 min. In terms of results, U/(min·g) FW was used.

The SOD activity was determined using test kits (Beijing Boxbio Science & Technology Co., Ltd., Beijing, China). When the xanthine oxidase coupling reaction system exhibited a 50% inhibition rate, the SOD activity in this system, denoted as U/g FW, was designated as a single enzyme activity unit.

### 2.7. Determination of Phenylalanine Ammonialyase (PAL), Cinnamyl Alcohol Dehydrogenase (CAD), 4-Coumarate/CoA Ligase (4CL), Cinnamate 4-Hydroxylase (C4H), and Peroxidase (POD) Activities

The determination of the PAL content was carried out in accordance with the method described by Xue et al. [25] with a few adaptations. Okra pods powder (0.2 g) was extracted in 1 mL of pre-cooled 0.1 mol/L boric acid buffer solution (pH 8.8, comprising 40 g/L PVP, 2 mmol/L EDTA, and 5 mmol/L *β*-mercaptoethanol). After homogenizing the mixture in an ice bath, it underwent centrifugation for 20 min at 4 °C at 12,000× *g*. An enzyme solution was recovered from the supernatant. The reaction mixture contained 3 mL of 50 mmol/L boric acid buffer (pH 8.8), 0.5 mL of 20 mmol/L L-phenylalanine, and 0.5 mL of the enzyme solution; it was held at 37 °C for 10 min. The absorbance at 290 nm was recorded before and after the incubation at 37 °C for 1 h. One unit of absorbance increase was considered as 0.01 per hour. PAL activity was expressed in terms of U/(h·g) FW.

The assay of CAD was performed as described by Goffner et al. [26]. Briefly, okra pods powder (1.0 g) was homogenized in 0.1 mmol/L of phosphate buffer (3 mL, pH 6.25, containing 2% polyethylene glycol (*w*/*v*), 10 mmol/L mercaptoethanol and 10% PVPP) under ice bath condition. The supernatant was extracted using centrifugation. The reaction mixture (0.8 mL, containing NADP and p-cinnamic acid) was mixed with the supernatant (0.2 mL) and incubated for 30 min at 37 °C. Added 100 μL of 1 mol/L HCl quickly to terminate the reaction. The OD_340_ samples were recorded. The definition of enzyme activity was a 0.01 absorbance rise per minute. CAD activity was expressed as U/g FW.

The activities of 4CL and C4H were determined using test kits (Beijing Boxbio Science & Technology Co., Ltd., Beijing, China). There were some changes in the measurement of 4CL activity; the okra pod powder was raised to 0.3 g, and the reaction time was shortened to 5 min. The C4H activity was measured by similarly changing the okra pod powder to 0.3 g, and the reaction time was extended to 10 min. The instructions for the other steps were followed. U/mg FW was used to express the results.

The POD activity was determined as described by Hu et al. [27] with a few adaptations. Okra pods powder (0.1 g) was homogenized with 1 mL of 0.1 mol/L acetic acid-sodium acetate buffer solution (including 1 mmoL PEG 6000, 4% PVPP, and 1% Triton X-100) under ice bath condition, and then centrifuged at 4 °C, 12,000× *g* for 20 min. The reaction mixture contained 0.5 mL of the supernatant and 3 mL of 25 mmol/L guaiacol solution, followed by the addition of 200 μL of 0.5 mol/L H_2_O_2_. The change in absorbance value of the POD enzyme at 470 nm was recorded. The results were expressed as U/g FW.

### 2.8. RNA Extraction and Sequencing

Okra pods stored by 0, 8, and 20 d were selected as transcriptome sequencing samples. The total RNA from okra was obtained utilizing the Trizol reagent kit (Invitrogen, Carlsbad, CA, USA), following the manufacturer’s instructions. Using an Agilent 2100 Bioanalyzer (Agilent Technologies, Palo Alto, CA, USA), the quality of RNA was evaluated. The cDNA libraries were sequenced on the Illumina novaseq 6000 platform by Genedenovo Biotechnology Co., Ltd. (Guangzhou, China). Low-quality data will interfere with subsequent analysis experiments, and low-quality raw reads should be filtered out. The fastp software (version: 0.18.0) was used to perform quality control on the raw reads to obtain clean reads. The clean reads were assembled using Trinity software (v2.8.4), and redundancy was removed to obtain Unigene.

### 2.9. Analysis of Sequencing Data

Differential expression of RNAs in the two groups of Okra was analyzed using DESeq2 software (v1.20.0). DEGs were defined as genes having a false discovery rate (FDR) < 0.05 and |log2FC| > 1 parameter.

All DEGs were annotated with Gene Ontology (GO) terms from the GO database (http://www.geneontology.org/, accessed on 18 November 2023), with Q-values ≤ 0.05 being significantly enriched GO terms compared to genomic background [28]. The DEGs were compared to the Kyoto Encyclopedia of Genes and Genomes (KEGG) database (http://www.genome.jp/kegg, accessed on 18 November 2023), with Q-values ≤ 0.05 being significantly enriched metabolic pathways compared to the genomic background [29].

### 2.10. Statistical Analysis

The experiments were replicated with 3 determinations for each test, and the results were reported as means ± standard deviation (SD). The statistical significance level (*p* < 0.05) was determined through a *t*-test analysis conducted using SPSS 20.0 software (SPSS Inc., Chicago, IL, USA).

## 3. Results

### 3.1. Effects of H_2_S on the Appearance of Okra Pods

**Figure 1:**
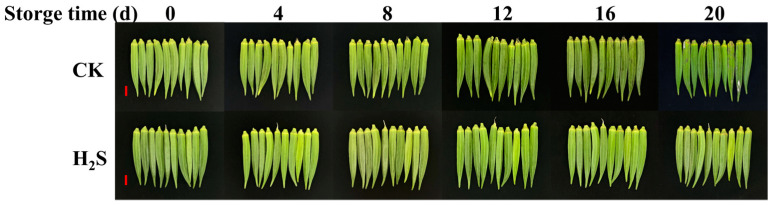
The appearance of okra pods in the control group and H_2_S-treated group during the storage process (0, 4, 8, 12, 16, 20 d). Stored at 5 ± 1 °C, 65–75% RH. Okra pods were fumigated with 0.5 mmol/L NaHS solution for 20 d. CK represents the control group, and H_2_S represents the H_2_S-treated group. Bar = 2 cm.

The appearance of okra pods under different treatment conditions is shown in Figure 1. As can be seen from the figure, the control group showed slight browning on 4 d, aggravated at 8 d, and mold spots were found at 12 d. A large amount of mold appeared in some okra pods at 20 d. Similar results were observed in Xiao et al. [30]. During the storage process, the okra pods treated with H_2_S were able to maintain their bright green color, and no mold was produced.

### 3.2. Effects of H_2_S on Weight Loss Rate and Hardness of Okra Pods

**Figure 2:**
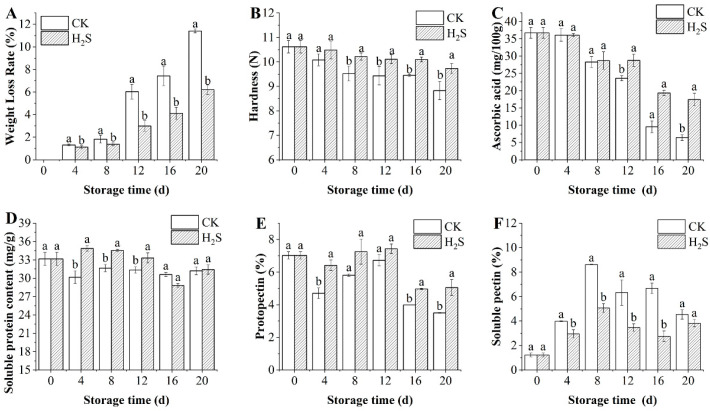
Effect of H_2_S on the rate of weight loss (**A**), hardness (**B**), ascorbic acid (**C**), soluble protein (**D**), protopectin (**E**), and soluble pectin (**F**) in postharvest okra pods during storage time. Different letters in the graph indicate significant differences between the H_2_S-treated group and the control (*p* < 0.05). CK represents the control group, and H_2_S represents the H_2_S-treated group. Data are expressed as mean ± SD.

The effect of H_2_S on the rate of weight loss of postharvest okra pods is shown in Figure 2A. The weight loss rate of postharvest okra pods was enhanced with the duration of storage due to respiration and transpiration of okra pods. The group treated with H_2_S significantly delayed the increase in the weight loss rate of okra pods compared to the control group (*p* < 0.05).

Hardness serves as a crucial index in assessing the storage quality of agricultural produce. The hardness of okra pods gradually decreased with increasing storage time and was always higher in the group treated with H_2_S than in the control (Figure 2B). From the 8th day of storage, the hardness of the H_2_S-treated group okra pods showed a significant difference compared to the control (*p* < 0.05), and its hardness was 1.07 times that of the control group. Overall, H_2_S fumigation could slow down the softening of okra pods.

### 3.3. Effects of H_2_S on the Contents of Ascorbic Acid, Soluble Protein, Protopectin, and Soluble Pectin in Okra Pods

The ascorbic acid content in okra pods was consumed during the ripening process and showed a decreasing trend (Figure 2C). The control group was significantly lower than the H_2_S-treated group from 12 d onwards. On day 20, the ascorbic acid content of the H_2_S-treated group was 17.45 ± 1.84 mg/g, which was 2.73 times higher than in the control batch (*p* < 0.05). According to Figure 2D, the soluble protein content of okra pods decreased after harvesting. However, the soluble protein of treated okra pods increased slightly and then decreased. The H_2_S-treated group increased to 34.86 ± 0.46 mg/g on the 4th day, a 1.16-fold rise in comparison to the control group. Therefore, H_2_S can increase the soluble protein content of okra pods throughout the early storage period, reduce the rate of decomposition of soluble protein, and delay senescence.

Pectin is composed of protopectin and soluble pectin. The content of protopectin was consistently higher in the H_2_S-treated group than in the control group (Figure 2E). On day 20, the content of protopectin in the control group was 3.50 ± 0.02%, and the H_2_S-treated group exhibited a 1.44-fold rise in comparison to the control group. The soluble pectin reached a peak at day 8 and then started to decline (Figure 2F). The results pointed out that H_2_S fumigation treatment could slow down the decline in pectin content and reduce the production of soluble pectin content in okra pods during storage.

### 3.4. Effects of H_2_S on the Contents of Lignin and Cellulose Content in Okra Pods

**Figure 3:**
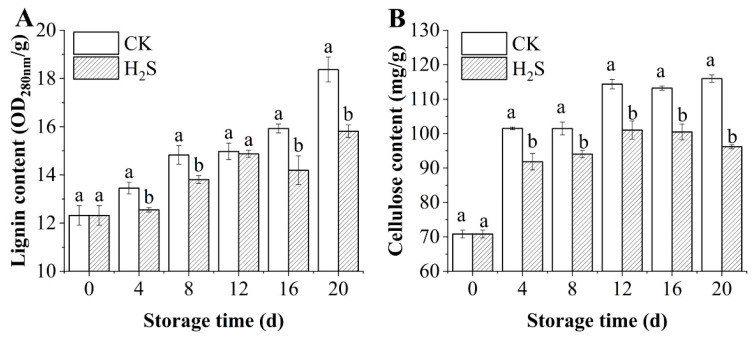
Effect of H_2_S on lignin (**A**) and cellulose content (**B**) in postharvest okra pods during storage time. Different letters in the graph indicate significant differences between the H_2_S-treated group and the control (*p* < 0.05). CK represents the control group; H_2_S represents the H_2_S-treated group. Data are expressed as mean ± SD.

Figure 3 illustrates the variations in lignin (A) and cellulose (B) content within okra pods throughout the storage duration. The lignin content of postharvest okra pods rose progressively during the period of storage, and the lignin content of the control batch increased from 12.32 ± 0.40 OD_280 nm_/g FW (0 d) to 18.37 ± 0.52 OD_280 nm_/g FW (20 d). Notably, the application of H_2_S inhibited the synthesis of lignin in postharvest okra pods. A similar situation existed for the cellulose content. The H_2_S-treated okra pods exhibited a lower cellulose content compared to the control, and cellulose content was significantly lower from day 4 (*p* < 0.05). At 20 d, the cellulose content in the control okra surpassed that of the H2S-treated batch by 1.21 times. Therefore, H_2_S may retard the lignification process by inhibiting the growth of lignin and cellulose in okra pods.

### 3.5. Effects of H_2_S on the Contents of H_2_O_2_, O_2_^·−^, and MDA in Okra Pods

**Figure 4:**
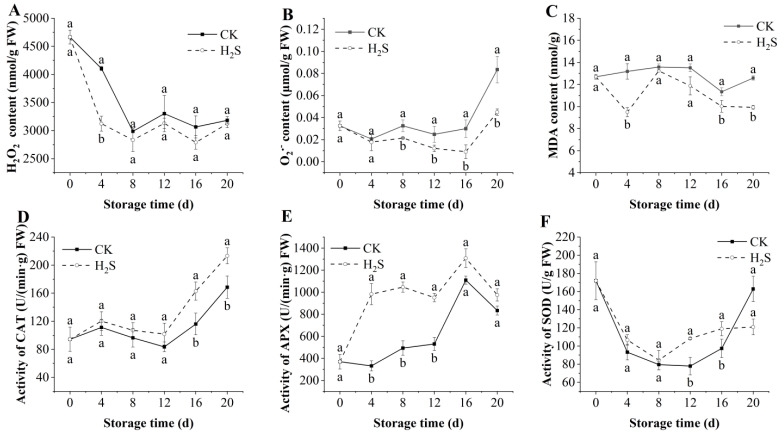
Effect of H_2_S on H_2_O_2_ (**A**), O_2_^·−^ (**B**), MDA (**C**), CAT (**D**), APX (**E**), SOD (**F**) in postharvest okra pods during storage time. Different letters in the graph indicate significant differences between the H_2_S-treated group and the control (*p* < 0.05). CK represents the control group; H_2_S represents the H_2_S-treated group. Data are expressed as mean ± SD.

During postharvest storage, okra pods naturally generate ROS, specifically H_2_O_2_ andO_2_^·−^, which contribute to tissue damage. The impact of H_2_S on the H_2_O_2_ level of postharvest okra pods is shown in Figure 4A. Throughout storage, the H_2_O_2_ content concentration steadily dropped, with the H_2_O_2_ content in the control group continuously being higher than that of the H_2_S-treated group. The results of Lv et al. [14] are in line with this tendency. During the storage process, the O_2_^·−^ content exhibited a gradual increase from the early and middle stages of storage, gradually increasing in late storage (Figure 4B). However, the control group always showed higher levels than the treated group. After 20 days of storage, the O_2_^·−^ content of okra pods enhanced from 0.033 ± 0.004 μmol/g to 0.084 ± 0.012 μmol/g in the control group, whereas the O_2_^·−^ content of the group treated with H_2_S was only 0.045 ± 0.003 μmol/g, which was significantly difference with control group (*p* < 0.05). MDA is a result of membrane lipid peroxidation. As illustrated in Figure 4C, the MDA content of the control group consistently exceeded that of the H_2_S-treated group. The results demonstrated that H_2_S treatment prevented the injury caused by MDA accumulation in the tissues, and the levels of H_2_O_2_ and O_2_^·−^ were decreased.

### 3.6. Effects of H_2_S on the Activities of CAT, APX, SOD in Okra Pods

The results of the changes in CAT, APX, and SOD of okra pods throughout the storage period are shown in Figure 4D–F. Throughout the whole storage time, the CAT activity showed a gradually increasing trend (Figure 4D), and the CAT activity in the group treated with H_2_S was significantly increased from the 16th day, which was higher than that in control okra (*p* < 0.05). Figure 4E illustrates trends in APX activity, which peaked on day 16 and then began to decline. The H_2_S treatment demonstrated the capacity to sustain elevated APX activity in okra pods. Notably, APX activity in the H_2_S treatment group began to be activated on day 4, which was 2.96 times that of the control batch. Figure 4F illustrates the effect of H_2_S on SOD activity. The activity reached its lowest point on the 8th day and then began to increase. The control and H_2_S-treated groups had similar trends, and higher activity was obtained after H_2_S fumigation.

### 3.7. Effects of H_2_S on the Activities of PAL, CAD, 4CL, C4H, and POD in Okra Pods

**Figure 5:**
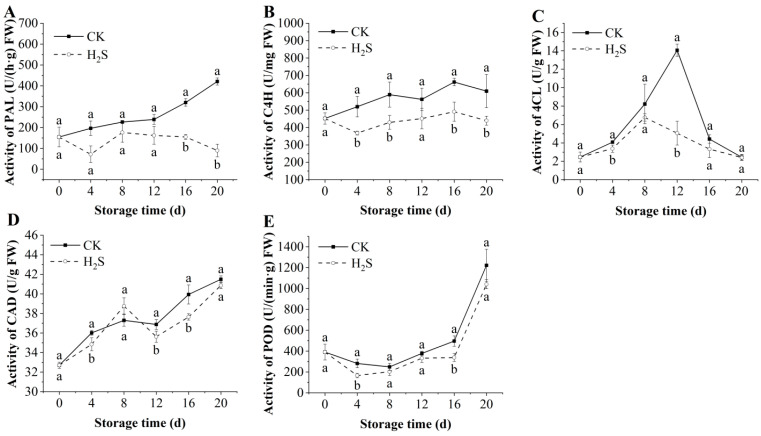
Effect of H_2_S on PAL (**A**), CAD (**B**), 4CL (**C**), C4H (**D**), and POD (**E**) in postharvest okra pods during storage time. Different letters in the graph indicate significant differences between the H_2_S-treated group and the control (*p* < 0.05). CK represents the control group, and H_2_S represents the H_2_S-treated group. Data are expressed as mean ± SD. FW = fresh weight.

To investigate the effect of H_2_S on the lignification of okra pods, the activities of PAL, CAD, 4CL, C4H, and POD in the pods were analyzed. The PAL activity of postharvest okra pods in the control group was consistently higher than that in the H_2_S-treated group (Figure 5A). H_2_S fumigation treatment was effective in reducing the PAL activity and inhibiting the synthesis of lignin in okra pods. A similar pattern was seen in the CAD activity of okra pods, which showed an increasing trend throughout the storage duration. As illustrated in Figure 5B, the CAD activity in the treatment group with H_2_S was demonstrably lower than that in the control sample at 4 d, 12 d, and 16 d (*p* < 0.05). The 4CL activity of okra pods treated with H_2_S exhibited an initial increase and then a subsequent decline trend (Figure 5C). This reached a peak on day 8, while the control group exhibited the highest value on day 12. During the storage duration, the 4CL activity of okra pods treated with H_2_S stayed consistently lower than that of the control sample. The C4H activity appeared to increase during the storage period, whereas the H_2_S-treated appeared to suppress this increase and maintain a more stable C4H activity from day 4 onwards (Figure 5D). On the 20th day of storage, the C4H activity of the H_2_S-treated group was 439.54 ± 26.07 U/mg, which was 27.89% lower than that of the control group (*p* < 0.05). The change in POD activity in the pre-storage period was relatively minor (Figure 5E). However, following the 12th day of storage, the POD activity of okra pods began to increase, with the greatest increase occurring between the 16th and 20th days of storage. It has been demonstrated that H_2_S can result in a reduction in POD activity in okra pods.

### 3.8. Transcriptome Profile of Postharvest Okra Pods RNA Libraries

A total of 15 transcriptome libraries (including three replicates) were obtained in this study, with an average of ~6.5 × 10^9^ bp CleanData produced for each group. The average Q20 and Q30 were 98% and 95%, and the distribution of GC content was between 43% and 45% (Supplementary Table S1). Following the assembly and de-redundancy of the sequencing data, 118,987 Unigenes were obtained, with a proportion of assembled bases of 40.2979%. These results indicate that the sequencing quality of okra pods transcriptome libraries met the criteria for subsequent expression analysis.

**Figure 6:**
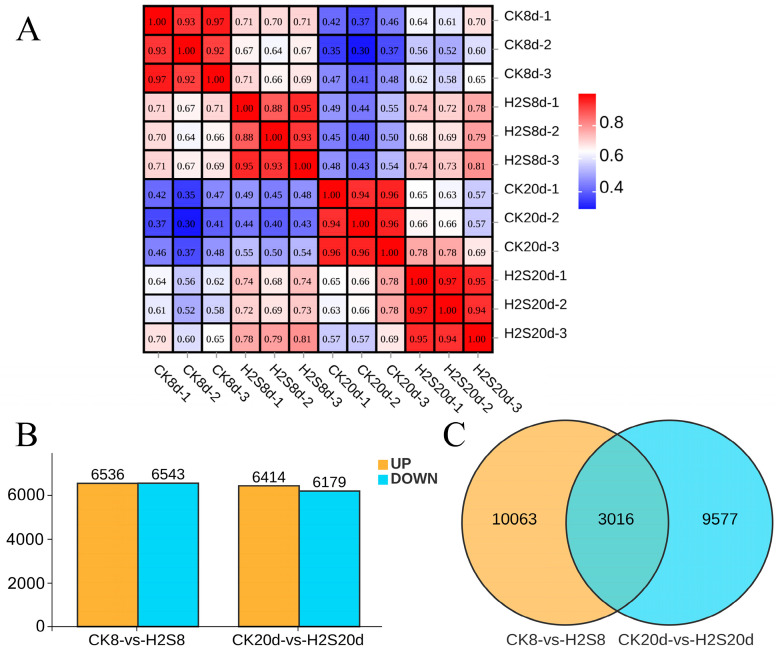
Correlations (**A**); scatter plot (**B**); Venn diagram (**C**) of DEGs between H_2_S-treated group and control. Red represents a positive correlation. Blue represents a negative correlation. Yellow represents up DEGs. Blue represents down DEGs. CK represents the control group, and H_2_S represents the H_2_S-treated group.

A heatmap of 12 samples is shown in Figure 6A. The Pearson correlation coefficient between the samples is greater than 0.8, indicating good reproducibility of the correlations among the samples. A total of 13,079 and 12,593 DEGs were obtained for CK 8 d-VS-H_2_S 8 d and CK 20 d-VS-H_2_S 20 d (Figure 6B). Compared to the control group, there were 6536 DEGs were up-regulated, 6543 DEGs were down-regulated in the H_2_S-treated group at day 8, 6414 DEGs were up-regulated, and 6179 DEGs were down-regulated at 20 d of storage. From the Venn diagram (Figure 6C), there were 3016 common DEGs shared between CK 8 d-VS-H_2_S 8 d and CK 20 d-VS-H_2_S 20 d.

### 3.9. Functional Enrichment of DEGs

**Figure 7:**
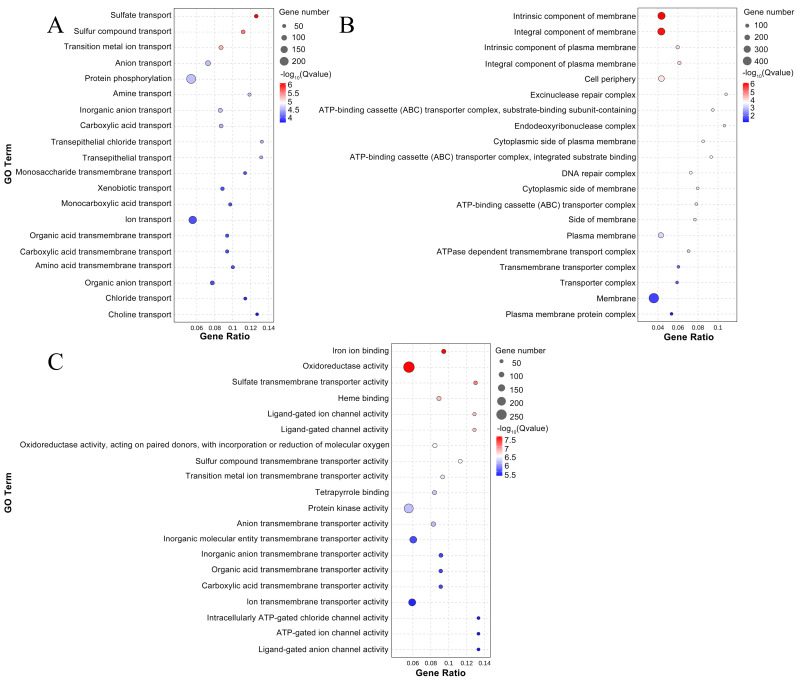
GO enriched top 20 bubble charts of target genes in okra pods: biological process (**A**); cellular component (**B**); molecular function (**C**). CK 8 d-VS-H_2_S 8 d and CK 20 d-VS-H_2_S 20 d were used as target genes. CK represents the control group; H_2_S represents the H_2_S-treated group.

DEGs shared by CK 8 d-VS-H_2_S 8 d and CK 20 d-VS-H_2_S 20 d were used as target genes for enrichment analyses. Figure 7 shows the top 20 GO terms with GO enrichment significance in the target genes, including biological process, cellular component, and molecular function. After removing redundancy, a total of 1495 DEGs were enriched in biological process (Figure 7A), with sulfate transport (36, 2.41%) and sulfur compound transport (39, 2.61%) being significantly enriched. A total of 912 DEGs were annotated to cellular components (Figure 7B), and the top two significantly enriched GO terms were intrinsic component of membrane (300, 32.89%) and integral component of membrane (293, 32.13%). A total of 1737 DEGs were annotated to molecular functions (Figure 7C); the analysis showed the significance of genes associated with iron ion binding (63, 3.63%), oxidoreductase activity (264, 15.20%), and sulfate transporter activity (35, 2.01%). The results indicate that DEGs after H_2_S treatment of okra pods are mainly related to oxidoreductase activity in molecular function.

**Figure 8:**
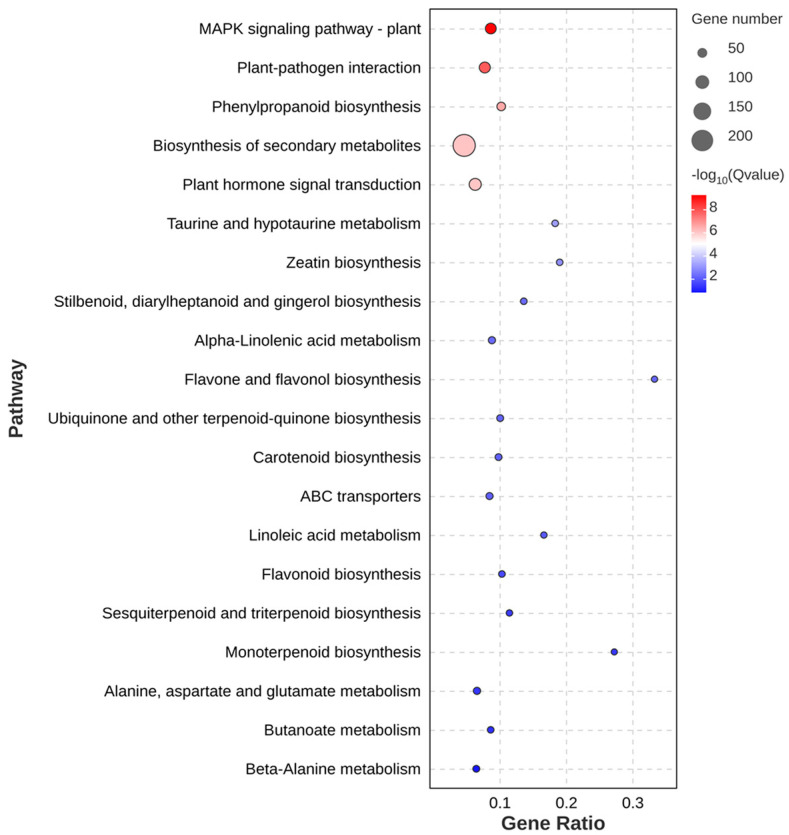
KEGG enriched top 20 bubble charts of target genes in okra pods. CK 8 d-VS-H_2_S 8 d and CK 20 d-VS-H_2_S 20 d were used as target genes. CK represents the control group, and H_2_S represents the H_2_S-treated group.

To obtain the metabolic pathways affected by the H_2_S treatment of okra pods, the Kyoto encyclopedia of KEGG pathways enrichment maps were constructed. The number of target genes with KEGG annotations was 635. According to the enrichment bubble plot (Figure 8), MAPK signaling pathway–plant (62, 9.76%), plant–pathogen interaction (64, 10.08%), and phenylpropanoid biosynthesis (34, 5.35%) were the top three metabolic pathways enriched with significance, followed by biosynthesis of secondary metabolites (205, 32.28%), plant hormone signal transduction (77, 12.13%) and taurine and hypotaurine metabolism (9, 1.42%). Three metabolic pathways obtained in KEGG enrichment analyses are important enrichment pathways in plant disease resistance [31,32]. The phenylpropanoid biosynthesis pathway is associated with lignification in okra pods [33]. The 34 DEGs are shown in Supplementary Table S2.

## 4. Discussion

Okra pod, known for its high nutritional value and green appearance, bears a resemblance to chili peppers. However, its susceptibility to water loss and wilting is heightened by its large surface area and harvesting in the summer [34]. Appearance is an important indication of fruit and vegetable quality. H_2_S treatment preserves the unchanged appearance of goji berry [9] and celery [35] and slows down the browning process [8]. The application of H_2_S also retarded the browning process and maintained the fresh appearance of okra pods. Hardness is closely related to pectin content. This study indicated that H_2_S treatment significantly reduced weight loss and showed higher protopectin and lower water-soluble pectin, which delayed softening and maintained the hardness of okra pods. As a result, the above were consistent with previous reports [36]. In addition, H_2_S treatment retarded nutrient loss, including ascorbic acid, soluble protein, and pectin, to prolong the storage period of fruit and vegetables [9,37]. We confirmed that H_2_S treatment leads to an increase in ascorbic acid content, thereby enhancing okra pods’ antioxidant capacity [14].

Redox metabolism plays a fundamental role in physiological processes; O_2_^·−^ and H_2_O_2_ are efficiently eliminated by antioxidant systems [38]. H_2_S can effectively reduce ROS levels, as evidenced by the findings of Deshi et al. [39] in lychee and Zhao et al. [12] in peach. The outcomes demonstrated that treating okra pods with H_2_S reduced the production of H_2_O_2_ and O_2_^·−^. APX stands as one of the pivotal enzymes involved in antioxidant systems. Treatment with H_2_S had a significant effect on ascorbic acid levels, rapidly increasing APX activity [14], showing lower H_2_O_2_ accumulation and higher ROS scavenging activity. It was discovered in the hawthorn study [34] that exposure to H_2_S could boost the activity of enzymes that scavenge ROS, accompanied by increased ascorbic acid buildup. Our experimental results demonstrated that H_2_S also strongly increased APX activity in okra pods during the pre-storage period, which was 2.12-fold in comparison to the control batch (8 d, *p* < 0.05). CAT is able to break down H_2_O_2_ into water and molecular oxygen. In this study, the CAT activity increased during the later stages of storage, facilitating the decomposition of H_2_O_2_ [40]. H_2_S treatment increased SOD activity to mitigate oxidative stress in tomato [37] and mushrooms [8]. Similarly, the same result was found in our study. It was presumed that H_2_S treatment could enhance the activity of enzymes in the antioxidant systems, with APX, CAT, and SOD playing a dominant role in different storage periods to avoid ROS buildup and preserve storage quality [14].

The lignification of fruits and vegetables is commonly related to the accumulation of lignin and cellulose [41]. Our results demonstrate that the group treated with H_2_S showed a beneficial impact on reducing the accumulation of lignin and cellulose. The significantly lower lignin content of the treatment group with H_2_S in comparison to the control sample may be attributed to the inhibitory influence of H_2_S on the activities of lignin synthesis-related enzymes. PAL is the key rate-limiting enzyme for lignin synthesis. This is consistent with previous findings that H_2_S treatment inhibits the lignification of pointed gourd by suppressing the activity of PAL [11], which has a similar effect on okra pods. Moreover, C4H, 4CL, and CAD equally act in the synthesis of lignin, and the increased activity of these enzymes will all contribute to the lignification process [42]. H_2_S dramatically reduced the activities of C4H, 4CL, and CAD in okra pods. Huang et al. [43] found that lignin synthesis was mainly correlated with the phenylpropanoid biosynthesis gene in navel oranges, and H_2_S treatment could significantly regulate the genes (*CitC4H, Cit4CL, CitCYP98A*, etc.) of the phenylpropanoid biosynthesis pathway. Sun et al. [44] reported that NO treatment could repress the genes of *AePAL*, *AeC4H*, and *Ae4CL* in okra pods and finally reduce the lignin content. In this study, a significant enrichment of 34 DEGs was observed within the phenylpropanoid biosynthesis pathway of the target genome, particularly *AePAL*, *Ae4CL1*, *AeCCOAOMT1*, *AeOMT*, *AeCYP84A1,* and *AeCYP73A12*, which were mainly connection with the enzyme activities of PAL, 4CL, and POD. H_2_S can down-regulate lignification-related enzyme activities by affecting the relevant genes in the phenylpropanoid biosynthesis pathway and, finally, delayed lignification in okra pods. Additionally, H_2_S maintained the visual quality and nutritional content, enhanced non-enzymatic antioxidants and antioxidant enzymes, and retarded the senescence of okra pods.

## 5. Conclusions

Numerous internal and external factors influence the ripening and senescence of fruits and vegetables after harvest. This study indicated that moderate H_2_S (0.5 mmol/L) effectively preserved the original appearance and internal nutrient (ascorbic acid, soluble protein, pectin) of okra pods, inhibited reactive oxygen species accumulation such as hydrogen peroxide and superoxide anion, and malondialdehyde -induced membrane lipid peroxidation, improved the activity of the antioxidant enzymes such as catalase, ascorbate peroxidase, and superoxide dismutase. Increased antioxidant activity helps maintain okra’s postharvest quality and retards aging. In particular, H_2_S treatment not only showed a 13.94% and 17.06% reduction in lignin and cellulose content, respectively, compared to the control, but also significantly inhibited the enzyme activities of lignin synthesis pathways, including phenylalanine ammonialyase, cinnamyl alcohol dehydrogenase, 4-coumarate:CoA ligase, cinnamate 4-hydroxylase, and peroxidase. Okra treated with exogenous H_2_S had a roughly 4-day longer shelf life than the control group. A total of 635 differentially expressed genes were obtained for the target genes in the Kyoto Encyclopedia of Genes and Genomes database enrichment analysis. Three metabolic pathways with the highest significance were MAPK signaling pathway–plant, plant–pathogen interaction, and phenylpropanoid biosynthesis, in which 34 differentially expressed genes were significantly enriched in phenylpropanoid biosynthesis. Additionally, exogenous H_2_S has the ability to postpone okra pod senescence processes and increase their shelf life. However, whether and how H_2_S is involved in the cross-linking of other hormones and signaling molecules (e.g., ethylene, abscisic acid, and nitric oxide) needs to be further elucidated. On the other hand, it is necessary to assess its safety and acceptable levels in fruit and vegetable storage and/or cooking, respectively, due to H_2_S causing a rotten egg flavor.

## Data Availability

The original contributions presented in the study are included in the article/Supplementary Materials; further inquiries can be directed to the corresponding author.

## Supplementary Materials

The following supporting information can be downloaded at: https://www.mdpi.com/article/10.3390/foods13162617/s1, Supplementary Table S1. Sequencing data statistics table. Supplementary Table S2. Thirty-four DEGs significantly enriched in the phenylpropanoid biosynthesis pathway in okra pods.
