# Supplementary material for: Hydrogen Sulfide Improves Postharvest Quality of Okra (Abelmoschus esculentus (L.) Moench) Pods by Enhancing Antioxidant Capacity and Delaying Lignification
Source: Foods. 2024 Aug 21;13(16):2617. doi: 10.3390/foods13162617 (PMC11353269; doi:10.3390/foods13162617)
Supplement: Supplementary file 1 [file foods-13-02617-s001.zip › foods-3128478-supplementary.pdf]

Table S1 Sequencing data statistics table

Sample	RawData(bp)	CleanData(bp)	AF_Q20(%)	AF_Q30(%)	AF_GC(%)
CK0d-1	6265505400	6147054023	6067058964 (98.70%)	5908041577 (96.11%)	2751306782 (44.76%)
CK0d-2	6448415400	6316386895	6236575164 (98.74%)	6077497638 (96.22%)	2820962542 (44.66%)
CK0d-3	5948686200	5833899105	5758212322 (98.70%)	5607036471 (96.11%)	2600717199 (44.58%)
CK8d-1	6096572700	5991726915	5912947582 (98.69%)	5754270313 (96.04%)	2664667719 (44.47%)
CK8d-2	7889138400	7746572841	7574568907 (97.78%)	7258002054 (93.69%)	3432120344 (44.31%)
CK8d-3	5996760300	5879435968	5787376610 (98.43%)	5605772281 (95.35%)	2611516710 (44.42%)
H_2S8d-1	7374867300	7232324818	7082147689 (97.92%)	6802133119 (94.05%)	3170918494 (43.84%)
H_2S8d-2	7722063900	7604510423	7448344407 (97.95%)	7162494482 (94.19%)	3346830946 (44.01%)
H_2S8d-3	5601833700	5492165998	5420146971 (98.69%)	5277446252 (96.09%)	2432376424 (44.29%)
CK20d-1	7411515600	7287095410	7135742682 (97.92%)	6850621366 (94.01%)	3264952856 (44.80%)
CK20d-2	5397249000	5305158686	5220483017 (98.40%)	5055312197 (95.29%)	2400068469 (45.24%)
CK20d-3	7675431600	7556896088	7403923634 (97.98%)	7123655706 (94.27%)	3369658610 (44.59%)
H_2S20d-1	5976842100	5877273165	5801469998 (98.71%)	5649998621 (96.13%)	2611474637 (44.43%)
H_2S20d-2	5789565300	5680306806	5600727990 (98.60%)	5442473576 (95.81%)	2516800638 (44.31%)
H_2S20d-3	7926273300	7809954195	7644720299 (97.88%)	7339791599 (93.98%)	3462674639 (44.34%)

Table S2 34 DEGs significantly enriched in the phenylpropanoid biosynthesis pathway in okra pods

symbol	description	symbol	description
AeCYP98A2	putative p-coumarate 3-hydroxylase	*AeGSVIVT00037159001*	PREDICTED: peroxidase 5-like
AeCYP98A2	putative p-coumarate 3-hydroxylase	*Ae4CL*	4-coumarate:CoA ligase 1-like
AeCYP98A2	hypothetical protein J1N35_042515	*AeAt1g30760*	berberine bridge enzyme-like 15
AePER42	peroxidase 42-like	*AePER20*	hypothetical protein ES319_D12G237100v1
--	caffeoylshikimate esterase-like	*AePAL1*	phenylalanine ammonia-lyase
AeCCOMT	hypothetical protein CXB51_035838	*Ae4CL1*	4-coumarate:CoA ligase 1-like
AeGSVIVT00037159001	peroxidase 5-like	*AePAL1*	phenylalanine ammonia-lyase
AeMEE23	reticuline oxidase-like protein-like	*AeOMT1*	caffeic acid 3-O-methyltransferase-like
AePAL	phenylalanine ammonia-lyase-like	*AePER53*	hypothetical protein CXB51_028399
AepoxN1	peroxidase N1-like	*AeCYP73A12*	cytochrome P450 CYP73A100-like
AeCAD9	probable cinnamyl alcohol dehydrogenase 9	*AeACT*	hypothetical protein CXB51_003207
AeCYP84A1	cytochrome P450 84A1-like	*AeCYP73A13*	trans-cinnamate 4-monooxygenase
AeCYP73A16	putative trans-cinnamate 4-hydroxylase	*AeCCR1*	putative cinnamoyl-CoA reductase
Ae4CLL6	4-coumarate--CoA ligase-like 6	*AeOMT*	caffeic acid 3-O-methyltransferase-like
Ae4CLL6	4-coumarate--CoA ligase-like 6	*AePAL*	phenylalanine ammonia-lyase
AePOD	peroxidase A2-like	*AeCCOAOMT1*	caffeoyl-CoA O-methyltransferase
AePOD	peroxidase A2-like	*Ae4CL1*	hypothetical protein ES332_D10G056800v1
